# Dimensionality of ICA in resting-state fMRI investigated by feature optimized classification of independent components with SVM

**DOI:** 10.3389/fnhum.2015.00259

**Published:** 2015-05-08

**Authors:** Yanlu Wang, Tie-Qiang Li

**Affiliations:** ^1^Department of Clinical Science, Intervention and Technology, Karolinska InstituteStockholm, Sweden; ^2^Unit of Medical Imaging, Function, and Technology, Department of Medical Physics, Karolinska University HospitalHuddinge, Sweden

**Keywords:** magnetic resonance imaging, functional neuroimaging, independent component analysis, pattern classification, machine learning, signal processing, image processing

## Abstract

Different machine learning algorithms have recently been used for assisting automated classification of independent component analysis (ICA) results from resting-state fMRI data. The success of this approach relies on identification of artifact components and meaningful functional networks. A limiting factor of ICA is the uncertainty of the number of independent components (NIC). We aim to develop a framework based on support vector machines (SVM) and optimized feature-selection for automated classification of independent components (ICs) and use the framework to investigate the effects of input NIC on the ICA results. Seven different resting-state fMRI datasets were studied. 18 features were devised by mimicking the empirical criteria for manual evaluation. The five most significant (*p* < 0.01) features were identified by general linear modeling and used to generate a classification model for the framework. This feature-optimized classification of ICs with SVM (FOCIS) framework was used to classify both group and single subject ICA results. The classification results obtained using FOCIS and previously published FSL-FIX were compared against manually evaluated results. On average the false negative rate in identifying artifact contaminated ICs for FOCIS and FSL-FIX were 98.27 and 92.34%, respectively. The number of artifact and functional network components increased almost linearly with the input NIC. Through tracking, we demonstrate that incrementing NIC affects most ICs when NIC < 33, whereas only a few limited ICs are affected by direct splitting when NIC is incremented beyond NIC > 40. For a given IC, its changes with increasing NIC are individually specific irrespective whether the component is a potential resting-state functional network or an artifact component. Using FOCIS, we investigated experimentally the ICA dimensionality of resting-state fMRI datasets and found that the input NIC can critically affect the ICA results of resting-state fMRI data.

## Introduction

Independent component analysis (ICA) is a data-driven, unsupervised analysis method for extracting resting-state functional connectivity networks (RFNs) (Calhoun et al., 2001; Beckmann et al., 2005; Kiviniemi et al., 2009; Schopf et al., 2010). Although ICA has been widely used for the analysis of resting-state fMRI data, there are still three inter-related issues that needs to be addressed: (1) The lack of gold standard or generally accepted RFN template; (2) the influence of the input number of independent components (NIC) or dimensionality on the ICA results; (3) the removal of artifact contaminated components. Manual classification of meaningful RFNs from the ICA results is currently a tedious but necessary step when conducting ICA of resting-state fMRI data, because only some of the independent components (ICs) represent meaningful RFNs associated with spontaneous neuronal activities, while other ICs reflect the effects of artifact contamination due to head motion, other physiological activities, and instrument imperfection. The manual evaluation of ICA results usually rely on visual inspection of the spatial patterns and their corresponding time courses of the of ICs (Bartels and Zeki, 2005). As summarized recently by Allen et al. (2011), the empirical criteria to select RFNs from ICs are based on the expectations that RFNs should exhibit peak activations in gray matter, low spatial overlap with known vascular, ventricular, motion, and susceptibility artifacts, and dominated by low frequency fluctuations below 0.08 Hz (Cordes et al., 2000). This way of manually selecting a subset of ICs as RFNs is not only biased, but also cumbersome, particularly when NIC is relatively large.

As summarized in Table 1, earlier studies aimed to develop automated classification of ICA results relied on relatively simple metrics from the time courses and spatial template matching. Perlbarg et al. (2007) used a step-wise regression approach and features based on the spatial and temporal patterns of the physiological noise to group ICs into noise and signal. Calhoun et al. (2005) utilized a brain atlas to sort ICs, which requires strong a priori knowledge on the spatial patterns of the activation. Sui et al. (2009) employed spatial criterion to automatically classify ICs. Their method relied on generating accurate cerebrospinal fluid (CSF) and gray matter masks. Kundu et al. (2012) conducted classification using the TE dependence of ICs, which seemed to be robust but requires the acquisition of multi-echo fMRI data.

**Table 1:** **Summary of previous studies on automated classification of ICA results**.

**Reference**	**Algorithm**	**Features**	**Applicability**	**Performance**
Perlbarg et al., 2007	Stepwise regression	Physiological noise, movement parameters	Individual	Mean sensitivity 0.87
Sui et al., 2009	Adaptive threshold	Spatial features, templates	Group, task-based fMRI	Mean accuracy 0.91
Douglas et al., 2011	Random Forest, AdaBoost, Naïve Bayes, J48 Decision Trere, K^*^, SVM	Unknown	Individual, task-based fMRI	Accuracy rates 0.92, 0.91, 0.89, 0.87, 0.86, 0.84 respectively
Kundu et al., 2012	Multiple regression	TE-dependency, R2^*^	Group and Individual, Multi-Echo EPI	Effective at detecting motion and pulsation artifacts. Denoised datasets show higher *t*-values in their connectivity maps.
Bhaganagarapu et al., 2013	k-means clustering	4 features, spatial and temporal	Group and Individual	Accuracy 0.997; Sochat et al., 2014 reports **(Individual)** Sensitivity 0.52, Specificity 0.89, **(group)** Sensitivity 0.42, Specificity 0.91,
Xu et al., 2014	Decision Tree	4 features, Spatial	task-based and resting-state fMRI with PET	Sensitivity 0.991, Specificity 1
Salimi-Khorshidi et al., 2014	SVM	>180 features	Individual	Accuracy 0.98 (multi-band EPI), 0.95 (standard EPI)
Sochat et al., 2014	Logistic Regression	246 features, spatial and temporal	Group and Individual	**(Individual)** Sensitivity 0.91, Specificity 0.91, **(Group)** Sensitivity 0.91, Specificity 0.81
Current study	SVM	5 features, spatial and temporal	Group and Individual	**(Individual)** Accuracy 0.91; **(Group)** Accuracy 0.99

In a number of more recent studies (see Table 1), automatic techniques based on more sophisticated machine learning algorithms has been applied to assist in grouping ICs into artifacts and potential RFNs. Douglas et al. (2011) compared the classification performances of six different machine learning algorithms ranging from K-star to support vector machine (SVM) using time course features. Xu et al. (2014) attempted to address the mechanistic motive and generalizability of automated classification of ICA results by incorporating information from head mask, auxiliary physiological recordings and PET activation results. The FIX plug-in (Griffanti et al., 2014; Salimi-Khorshidi et al., 2014) for FSL package achieved high accuracy classification of single-subject ICA results by employing ensemble learning based on multi-level classifiers and a large number of features. Sochat et al. (2014) used even a more comprehensive pool of temporal and spatial features (up to 246) to perform automated classification of ICs but attained lower accuracy than FSL-FIX. On the other hand, Bhaganagarapu et al. (2013) accomplished robust classification of ICA results for both group and single-subject data using a method based on k-means clustering of four features associated with the smoothness, edge activity, and temporal frequencies of the ICs. Overall, the progress in improving the accuracy of the automated classification of ICs has been quite promising. It becomes feasible to test automated classification for systematic studies involving a large of ICs.

ICA results for resting-state fMRI are sensitive to the specified NIC or the dimensionality. Neither the true numbers of RFNs in the data nor the degree of artifact contamination is known *a priori* for a given resting-state fMRI dataset. The effects of input NIC on ICA results can be systematically studied by evaluating the number of RFNs as a function of the input number of components in combination with tracking the changes in the specific ICs (Abou Elseoud et al., 2010, 2011; Elseoud et al., 2011). Different methods, such as, AIC, Minimum description length (MDL), and probabilistic principle component analysis (PPCA) have been proposed to model the noise characteristics and estimate intrinsic dimensionality of resting state fMRI data (Cordes and Nandy, 2006). However, uncorrelated noise models like Akaike information criterion (AIC), MDL, and PPCA tend to over-estimate the dimensionality for fMRI data (Li et al., 2007; Xie et al., 2009). In practice, the numbers of ICs used in different studies vary widely, which makes it difficult to directly compare the RFN results from the different studies.

The purpose of this study is two-fold: (1) Develop a robust tracking and binary sorting framework based on feature optimized classification of ICs with SVM (FOCIS) techniques to reduce some of the limitations overviewed above; (2) Use the developed method to investigate how the extracted RFNs are influenced by the selection of NIC. As discussed above, optimal feature selection is not only important for improving computation efficiency, but also very critical for improving classification accuracy and general applicability through reducing model complexity which makes it less likely to over-fit. We carefully selected relatively few features based on their explanation power (*p* < 0.01) to maximize the AIC to avoid over-determination. This was done to maximize the general applicability of FOCIS in cross-dataset classification. The focus of our study was the classification of group ICA results. The manual and automated classifications of the group ICA results were carried out after t-score and cluster size filtering were conducted to the one-sampled t-score map to ensure a statistical significance *p* > 0.01.

We tested the FOCIS framework on a dataset acquired by us and 6 other datasets acquired at different sites, which are openly accessible. The study included also a formal performance evaluation through direct comparison with the published FSL-FIX package. Another important aspect of our study is to use the developed framework to investigate systematically how the selection of NIC affects the ICA results. Our results indicate that the ICA dimensionality is far from a resolved issue. Therefore, we also implemented a module in the FOCIS framework to facilitate automatic tracking of a given IC component as a function of NIC. This enabled us to study how a given IC component changes with increasing NIC.

## Materials and methods

### Ethics statement

The Central Ethical Review Board in Stockholm Province, Sweden approved the ethical permission application for this study. The study permission included the consent form used to provide information and obtain consent. All participants provided informed consent by voluntary signature.

### Resting-state fMRI data

Seven resting-state fMRI datasets from normal volunteers were used for the development and validation of the framework. We acquired one dataset ourselves by using a 3T whole-body clinical MRI scanner (TIM Trio, Siemens Healthcare, Erlangen, Germany) with a 2D gradient-recalled echo echo-planar imaging technique. The other 6 datasets were downloaded from an open-access database (https://www.nitrc.org/frs/?group_id=296). More details of the data acquisition parameters and the demographics of the participants are summarized in Table 2.

**Table 2:** **Acquisition parameters for seven the resting-state fMRI datasets and subject demographic information**.

**Dataset**	**Source**	**Male/Female**	**Age**	**Time-frames**	**Slice thickness**	**Slices**	**TR**	**FOV**	**Matrix size**
0	Our own	40/46	21–84	300	3.6	32	2	220	64 × 64
1	TRT[1]	15/11	13–29	197	3	34	2	192	64 × 64
2	1636[2]	13/15	23–44	195	4	34	2.3	192	64 × 64
3	1624[2]	16/21	20–42	195	4	47	2.3	192	64 × 64
4	1600[2]	8/15	20–40	123	3	39	2.5	256	96 × 96
5	2085[2]	10/15	22–49	197	3	36	2	192	64 × 64
6	1616[2]	12/12	20–71	115	4	32	2	220	64 × 64

[1] http://www.nitrc.org/projects/nyu_trt/

*[2] https://www.nitrc.org/frs/downloadlink.php/<Datasource> (where <Datasource> is the number in the Source column where the footnote is referenced)*.

### Preprocessing

The resting-state fMRI datasets underwent the same preprocessing procedure, which were performed with AFNI (http://afni.nimh.nih.gov/afni) and FSL (http://www.fmrib.ox.ac.uk/fsl) programs with a bash wrapper shell (Wang and Li, 2013). The first 10 timeframes in each data set were removed to ensure signal steady state. After temporal de-spiking, six-parameter rigid body image registration was performed for motion correction. The average volume for each motion-corrected time series was used to generate a brain mask to minimize the inclusion of the extra-cerebral tissues. Spatial normalization to the standard MNI template was performed using a 12-parameter affine transformation and mutual-information cost function. During the spatial normalization the data was also resampled to isotropic resolution using a Gaussian kernel with FWHM = 4 mm. Nuisance signal removal was achieved by voxel-wise regression using the 14 regressors based on the motion correction parameters, average signal of the ventricles and their 1st order derivatives. After baseline trend removal up to the third order polynomial, effective band-pass filtering was performed using low-pass filtering at 0.08 Hz. Local Gaussian smoothing up to FWHM = 6 mm was performed using an eroded gray matter mask (Jo et al., 2010).

### Independent component analysis

The lowest value that we can specify for NIC is 2 in ICA of resting-state fMRI data. With adequate preprocessing, ICA at NIC = 2 produces typically two large networks: one corresponding to the motor-sensory network combined with the visual network and the other corresponding to a cognitive functional network somewhat resembles the default mode network (DMN). The visual network usually become the next split-off at NIC = 3. With NIC > 3, the precise ICA representation of a resting-state fMRI dataset depends on the noise characteristics of the dataset. Defining a basic set of RFNs and artifacts is an extremely difficult and complex task beyond the scope of this investigation (Wig et al., 2011, 2014; Schultz et al., 2014). However, consensus from multiple studies suggests that there is a relatively stable set of RFNs that provide an appropriate description of the functional connectivity hierarchy at the level of major brain networks. For example, the 10 consistent across-subject RFNs from Damoiseaux et al. (2006), the 10 task and resting-state matched networks from Smith et al. (2009), and the 7-network parcellation from Yeo et al. (2011). Assuming a need for at least twice as many components as the 10 potential RFNs to allow for the adequate modeling of both RFNs and artifact components associated with noise and physiological sources, Schultz et al. (2014) created a 20 component ICA template and used it for template-based ICA of resting-state fMRI data. Along similar line of thinking, we assume that it is necessary to use at least a NIC = 20 to extract a complete set of RFNs with group ICA from a resting-state fMRI dataset contaminated with artifacts and focus our investigation of ICA dimensionality on NIC = 20. Since the results from previous ICA of fMRI data with NIC up to 90 indicated that performing ICA in an unnecessarily high dimensional subspace decreases the stability of the algorithm and degrades the integrity of the ICA representation of functional networks in the brain, we choose NIC = 100 as the upper limit in our investigation.

Group ICA was performed on the resting-state fMRI datasets 0–5 using the standalone *melodic* program in FSL package (Smith et al., 2004; Woolrich et al., 2009; Jenkinson et al., 2012). The analysis option of multi-session temporal concatenation was selected to extract common spatial patterns without assuming the consistent temporal response pattern across the subjects (Beckmann and Smith, 2004). By default *melodic* can estimate the dimension of the input data by performing a Bayesian analysis and use it for ICA. The estimated dimensions for the 7 datasets varied from 21 to 38. To study how the ICA results are influenced by the specification of input NICand test the stability of the SVM-based framework, besides ICA at the estimated NIC, 80 additional group ICA runs were also carried out for the resting-state fMRI dataset 1–5 by systematically increasing NIC from 20 to 100. Single subject ICA was also performed for the first 5 subjects included in the dataset 6 with NIC = 30. When NIC was explicitly specified as an input to *melodic*, the estimated default NIC by the Bayesian analysis should not affect the ICA results.

Before automated classification of the ICs with FOCIS and FSL-FIX, the one-sampled *t*-test maps for the ICs from the group ICA were first assessed by setting an uncorrected voxel-wise threshold at *p* < 0.001 and the minimum voxel cluster size of 20 contiguous voxels. The probability of random field of noise producing a cluster of size ≥20 was estimated at *p* < 0.01. This was assessed by Monte-Carlo simulation result obtained from the AFNI program, *AlphaSim*, with the following main input parameters: FWHM = 6.2 mm estimated average by running 3dFWHM on the input data to ICA, voxel-wise threshold value *p* < 0.001, and 5 × 10^5^ iterations.

### Manual evaluation of IC maps

Group ICA results for the dataset 1 at NIC = 50 was chosen as the training input for both FOCIS and FSL-FIX, the ICA results for datasets 1–6 at NIC = 30, and dataset 0 at NIC = 70 were all manually classified for cross-validation. In addition, for self-verification purpose, the group ICA results for dataset 1 at NIC = 70 and 90 were also manually classified. The results of manual classification were used to test the precision, accuracy, sensitivity, and specificity of the classification model. The ICA results were manually examined by at least two experienced neuroscientist raters and were classified into two broad classes: Potential RFN (class RFN) or artifacts (class ART). The classification criteria for the manual evaluation can be approximately summarized as the following:

1. A potential RFN has to fulfill the voxel-wise threshold *p* < 0.001 and the minimum cluster size >20.
2. A potential RFN exhibits peak activation in cortical gray-matter.
3. A potential RFN possesses little spatial overlap with known vascular, ventricular, motion, and susceptibility artifacts.
4. The associated time course for a potential RFN should reflect the expected low frequency spontaneous fluctuations with adequate dynamic range.
5. The geometry of an involved region of interests in a potential RFN is reasonably compact and smooth instead of extreme shape.

The overall classification scheme for class ART was more confined by using high cost for it to ensure that the potential RFNs are not discarded as artifacts. Whenever there exists uncertainty or inconsistency between the evaluation results from two reviewers, the involved ICs were classified into RFN class. This may increase the false negative rate in the classification results. However, this is appropriate given our goal of not discarding potential RFNs.

### Feature selection

A total of 18 initial features were devised to reflect the criteria used for visual inspection. These features include measures for spatial patterns, time-course characteristics, and spectral information. The descriptions for the initially devised features are provided in Appendix A (Supplementary Material) in more details including the relevant mathematical definitions. Calculations of the features were implemented in R in combination with AFNI programs, which are detailed in Appendix B (Supplementary Material). All features were conditioned prior to use in feature selection, training, and classification. This involves mean-centering (removal of mean) and scaling (division by standard deviation). All features were tested for explanatory power on the training dataset using a general linear model with a binomial variance function and a logit link function. We used F-score to select the features with high significant explanatory power (*p* < 0.01) out of the initial 18-item feature space. The procedure is summarized below:

1. Calculate F-score of each feature.
2. Manually pick a relative low F-score threshold to drop features with F-score below the threshold.
3. Randomly split the training data into training and validation subsets.
4. Predict the validation subset using SVM procedure based on the model built with the training subset and high F-score features.
5. Repeat the steps 4 multiple times to achieve a steady average validation error.
6. Repeat the steps 2–6 by incrementing F-score thresholds to drop more features with relatively low F-scores until the validation accuracy decrease significantly.

As shown in Table 3, with the group ICA training datasets we settled down on a model with only 5 most significant features (*p* < 0.01). A model with fewer parameters is less complex and more likely to be biased. On the other hand, a simpler classification model with limited Vapnik–Chervonenkis dimension has higher computational efficiency and lower risk of over-fitting. Optimal feature selection is, therefore, very important for the construction of classification model.

**Table 3:** **Results of explanatory power test for the five most significant (*p* < 0.01) features**.

**Coefficients**	***t*-value**	***p*-value**
Positive *t*-value with gray-matter overlap	6.23	9.97e-07
Peak voxel location in gray matter	5.79	3.23e-06
Frequency ratio of IC time course	5.14	1.90e-05
1-lag autocorrelation of IC time course	3.60	0.12e-03
Cluster bounding box to voxel count ratio	3.38	0.21e-03

### Construction of the classification model

A boundary-constraint SVM C-classification model was constructed using the training datasets and the 5 most significant features with *p* < 0.01. The boundary-constraint SVM classification solves a variant of quadratic problem using modified TRON optimization software (Lin and Moré, 1999; Mangasarian and Musicant, 1999). The resulting feature space is 50 training points × 5 features. The binary classification model uses a non-linear radial basis function kernel and 100 in cost. This was chosen to penalize false positive rates more severely than false negatives to avoid misclassification of potential RFNs as artifacts.

The framework uses the R package *kernlab* (Karatzoglou et al., 2007) for its SVM implementations. *Kernlab's* SVM implementation is based on LIBSVM (Chang and Lin, 2011) which is a popular implementation of SVM. To solve the optimization problems encountered in the training of the kernelized SVM, LIBSVM uses a minimal optimization algorithm. Gaussian radial basis function (RBF) kernel is used as a kernel function for non-linear SVM classification (in contrast to the dot product for linear SVM). The kernel function between two samples ***x*** and ***y*** is defined as:

$$k(x,y)=\exp(\sigma ||x-y|{|}_{2}^{2})$$

where σ is a free parameter. Optimal values of σ is shown to lie between the 0.1 and 0.9 quantiles of the ||x-y||statistic (Caputo et al., 2002) and any value is within these quantiles leads to good performance (Karatzoglou et al., 2004). Therefore, the value of σ was taken to be a random value within the 0.1 and 0.9 quantiles, which were estimated by using a random subsample of the training dataset. The choices of parameters were confirmed by hyper-parameter optimization through an exhaustive grid searching in input space of the cost (C) and free parameter (σ) within the boundary constraints C = [10, 1000] and σ = [0.1, 1.0] with a grid size of ΔC = 10 and Δσ = 0.1. Cross validation by maximizing accuracy while retaining sensitivity to 1 was carried out during self-verification with dataset 1, NIC = 30, 50, 70, and 90. It was confirmed that the performance is maximized and insensitive to the choice of σ within the predefined quantiles 0.1–0.9 and C within 100–210.

**Figure 1:**
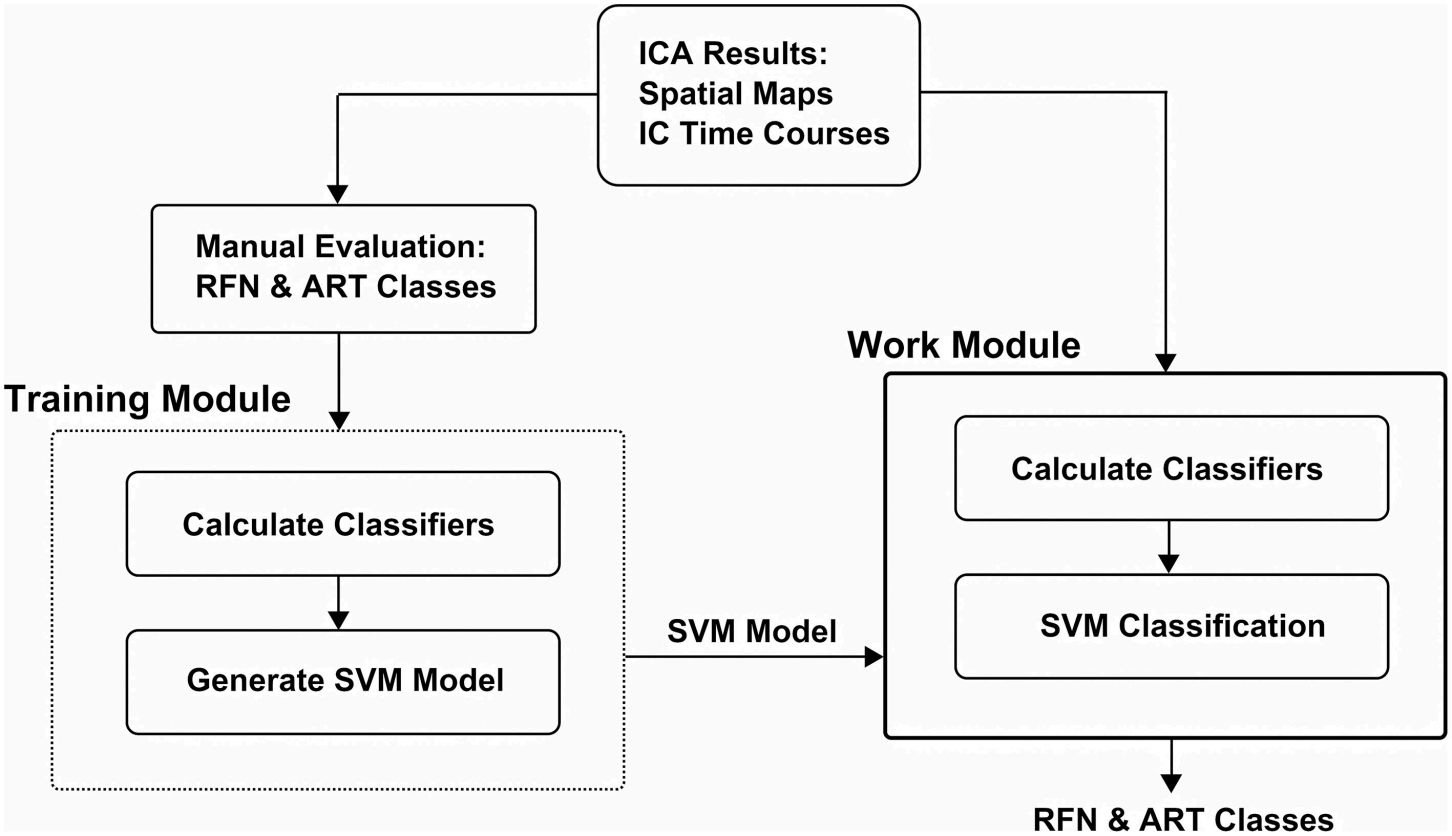
**Pipeline schematic of FOCIS framework**.

The workflow of the classification framework is illustrated in Figure 1. The training module takes ICA results (IC spatial map and IC time-course) as inputs to calculate the features. The calculated feature values with the manual inspection results are then used to generate a SVM classification model. The classifier module calculates the features from ICA results the same way as the training module. But it uses the calculated features together with the SVM classification model output from the training module to classify the ICs.

### Comparison between the automated and manual classifications

We conducted direct comparison of the classification performances between FOCIS and FSL-FIX by assuming the manual evaluation result as the ground truth. For direct comparison with FSL-FIX, we adapted the output results from group ICA so that automated classification can be performed using FSL-FIX, since FSL-fix was designed for single-session ICA, some required input parameters for FSL-FIX need to be compiled manually in order to use FSL-FIX for classification of the group ICA results. These included the concatenated 4D fMRI data for individual subject, the temporal mean of the 4D fMRI data, concatenated motion parameters, and the group average of transformation matrix. It should be pointed out that the outputs of the group and single subject ICA can be directly used for automated classification with FOCIS, because it requires no additional input except for the IC time course and spatial maps. The automated classification results from both FOCIS and FSL-FIX were then compared with those from manual evaluation. For a given IC, we verified if the automated classification is consistent with the manual evaluation. We also counted and compared the numbers of ICs in the RFN (N_RFN_) and ART (N_ART_) classes as determined by the automated and manual classifications. The most relevant performance index for automated classification here is the accuracy or the true negative rate (the percentage of true artifact component detected), since FOCIS was trained with heavy penalty on false positive rate. When a mismatch appeared between the automated and manual classification, the misclassified IC was further investigated to identify potential causes of the misclassification.

### The effects of input NIC on ICA results

We analyzed the datasets 1–5 with group ICA using a wide range of input NIC from 20 to 100. To understand how the ICA results are influenced by the selection of NIC, we investigated N_RFN_ and N_ART_ obtained from the automated and manual classifications as a function of NIC. We also investigated how the contribution of variance changed with NIC for some specific ICs. Furthermore, we constructed a regression module in FOCIS so that it can be used to track the changes of a given IC in a series of ICA results obtained from the same fMRI dataset by analyzing it with different NIC. The regression model was constructed using an epsilon (ε) support vector regression algorithm. Similar to SVM classification, the support vector regression algorithm only uses a subset of the training data (Vapnik et al., 1997). The cost function for building the model ignores any training data that is close (within a threshold ε) to the model prediction (Smola and Schölkopf, 2004). The regression model provides class probabilities as output instead of class labels. To track a specific IC in a series of ICA results, we constructed the model by labeling the specific IC as class 1 and all other ICs as class 0 using the ICA result with NIC = 30. When the SVM regression model was applied to ICA results with a larger NIC, we used the model to estimate class probabilities for all ICs. Then, we rank the ICs according to their class probabilities (the level of similarity to the specific IC). With the ranking, we can identify the corresponding IC in the ICA results of different NIC that matches closest to the given IC.

To quantify the changes in spatial patterns and the associated time courses of a given IC as a function of NIC we introduce a change index of spatial overlap and temporal association (CISOTA) which is defined in the following equation:

$$\begin{array}{l} \text{CISOTA = 1/}(\text{spatial overlap}\times \text{correlation coefficient of the IC}\\ \text{ time course}). \end{array}$$

where the spatial overlap for a given IC is defined as the fraction of overlap area relative to the original area of the spatial pattern. We evaluated CISOTA for three typical RFNs (motor-sensory, visual, and DMN) and three artifact components as function of NIC. Furthermore, we also evaluated the cross-sectional changes of all ICs when NIC was incremented by 1 at different NIC values.

## Results

### Feature selection

Amongst the 18 initially devised features, 5 are highly significant (*p* < 0.01) as determined by the explanatory power test (see Table 3). The model based on the subset of 5 parameters achieved a low AIC of 3.71 compared to AIC = 17.03 for the full model including 18 parameters. The relative AIC between the two models is $AIC_{rel}=\exp\left(\frac{3.71-17.03}{2}\right)=0.0013$, which indicate that the model based on the subset of 5 parameters is much likely to minimize the information loss than the full model (Akaike, 1974). To avoid over-fitting, therefore, we take no longer the full model into further consideration.

**Figure 2:**
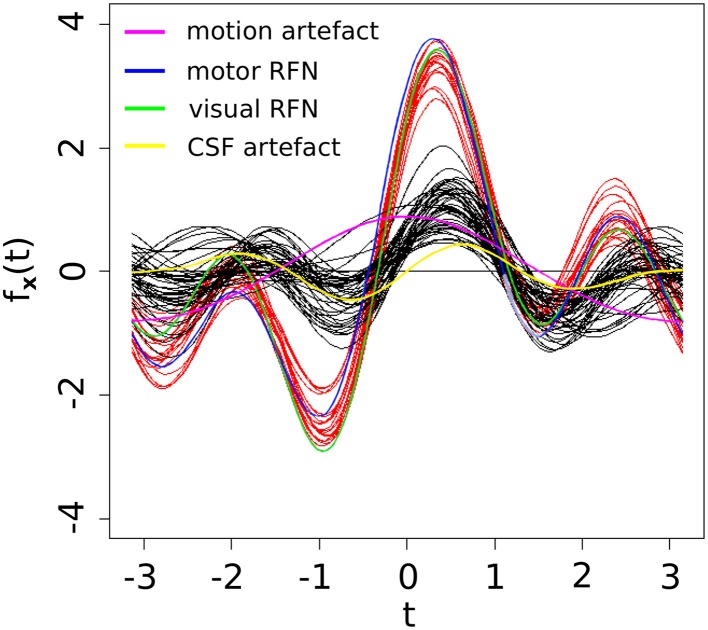
**Visually inspected ICs in feature space visualized using Andrew's curves**. Selected feature space consists of only the significant (*p* < 0.01) features. Red curves represent class RFN and black curves represent class ART.

As shown in Table 3, three of the significant features are associated with the spatial patterns of IC maps, whereas the other two features are related to the characteristics of IC time courses. Figure 2 depicts all the manually evaluated ICs used for training (dataset 1, NIC = 50) in the feature space of the five most significant features. All ICs in feature space is visualized as Andrews curves as a method to visualize high-dimensional data (Andrews, 1972). Each IC's data point in feature space ***x*** = [*x*_1_, …, *x*_5_] defines a curve through the function *f*(***x***, *t*) = *x*_1_*sin*(*t*)+*x*_2_*cos*(*t*)+*x*_3_*sin*(*2t*)+*x*_4_*cos*(*2t*)+*x*_5_*sin*(*3t*). This function is uniquely defined and plotted between −π and π. This formula can also be regarded as the projection of the data point onto the vector [*sin(t), cos(t), sin(2t), cos(2t), sin(3t)*].

### Construction of the classification model

**Figure 3:**
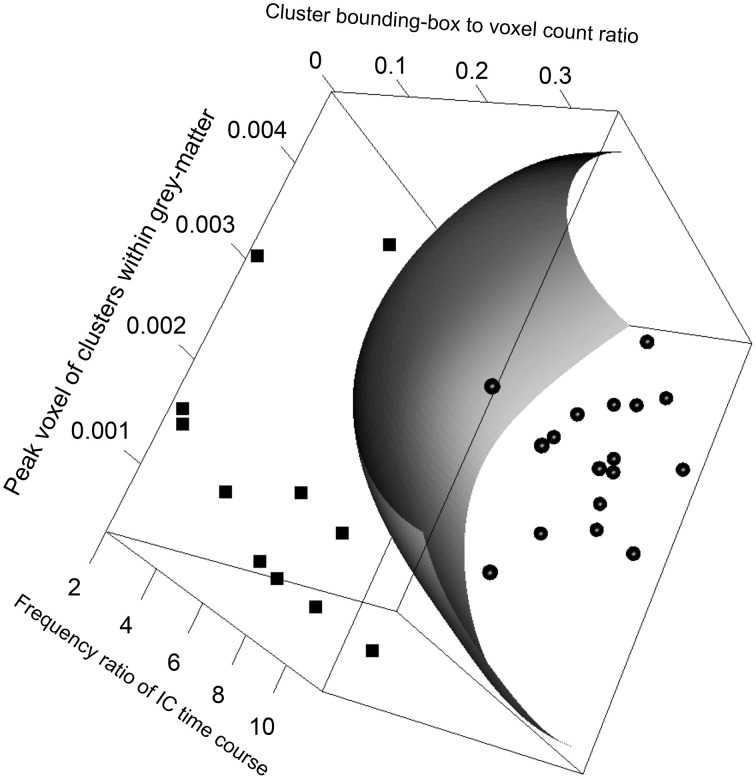
**Visualizing the FOCIS classification model as a dividing hyper-plane in 3D feature space projected onto its 3 dimensions with greatest variances**.

The SVM classification model constructed with the five most significant features described above was trained with the training dataset. The resulting model has 23 support vectors and the radial kernel size σ = 0.38. Self-verification of the model resulted in perfect classification with 100% in specificity and 100% in sensitivity for both the training dataset. Figure 3 illustrates the trained SVM model as a hyper-plane in a 3D projection of the 5D feature space. As illustrated in Figure 3, a hyper-plane of maximum variance separates appropriately the two classes of ICs in the training dataset.

### Comparison between the automated and manual classifications

The results from the automated and manual classifications are summarized in Tables 4–6. On average the automated classification based on FOCIS achieved zero false positive and 4/231 false negative rates in identifying artifact contaminated ICs of the group ICA results, signifying a 98.27% overlap between the FOCIS-based and human expert classifications in identifying artifact contaminated ICs (see Table 4). The false negative rates for FSL-FIX in identifying artifact contaminated ICs of the group ICA results is 17/231, corresponding to 92.64% overlap with the manual classification (see Table 4). As shown in Table 5, both FOCIS and FSL-FIX achieved robust classifications of the group ICA results. The performance of FOCIS in precision, accuracy and specificity is slightly higher than FSL-FIX when compared on the basis of using the identical training dataset of group ICA for dataset 1 at NIC = 50. As shown in Table 5, the accuracy of FOCIS in the automated classification of single subject ICA results is 91%, which is not as robust as that for the group ICA results.

**Table 4:** **Summary of the automated (FOCIS and FLS-FIX) and manual classification results for the group ICA results**.

		**FOCIS**		**Manual Evaluation**		**FSL-FIX**	
**Dataset**	**NIC**	**NART**	**NRFN**	**NART**	**NRFN**	**NART**	**NRFN**
0	70	29	41	30	40	30	40
	30	13	17	13	17	13	17
1	50	25	25	25	25	25	25
	70	39	31	39	31	37	33
	90	56	34	56	34	51	39
2	30	14	16	15	15	12	18
3	30	15	15	16	14	16	14
4	30	20	10	20	10	17	13
5	30	16	14	17	13	14	16

**Table 5:** **Precision, accuracy, specificity, and sensitivity of the automatic classifications based on FOCIS and FSL-fix frameworks for group ICA results**.

**Dataset**	**NIC**	**Precision**		**Sensitivity**		**Specificity**		**Accuracy**	
		**FOCIS**	**FLS-FIX**	**FOCIS**	**FLS-FIX**	**FOCIS**	**FLS-FIX**	**FOCIS**	**FLS-FIX**
0	70	0.97	1.00	1.00	1.00	0.98	1.00	0.99	1.00
	30	1.00	1.00	1.00	1.00	1.00	1.00	1.00	1.00
1	50	1.00	1.00	1.00	1.00	1.00	1.00	1.00	1.00
	70	1.00	0.87	1.00	1.00	1.00	0.86	1.00	0.93
	90	1.00	0.89	1.00	1.00	1.00	0.83	1.00	0.93
2	30	0.94	0.80	1.00	1.00	0.93	0.83	0.97	0.90
3	30	1.00	1.00	1.00	1.00	1.00	1.00	1.00	1.00
4	30	1.00	0.85	1.00	1.00	1.00	0.77	1.00	0.90
5	30	0.94	0.82	1.00	1.00	0.93	0.81	0.97	0.90
Mean		0.98	0.92	1.00	1.00	0.98	0.90	0.99	0.95

**Table 6:** **Precision, accuracy, specificity and sensitivity of the automatic classification with FOCIS for single subject ICA results**.

**Subject**	**NIC**	**Precision**	**Sensitivity**	**Specificity**	**Accuracy**
1	30	0.93	1.00	0.94	0.97
2	30	0.69	0.90	0.80	0.83
3	30	0.78	0.78	0.90	0.87
4	30	1.00	1.00	1.00	1.00
5	30	0.85	0.92	0.89	0.90
Mean		0.85	0.92	0.91	0.91

### The effects of input NIC on ICA results

**Figure 4:**
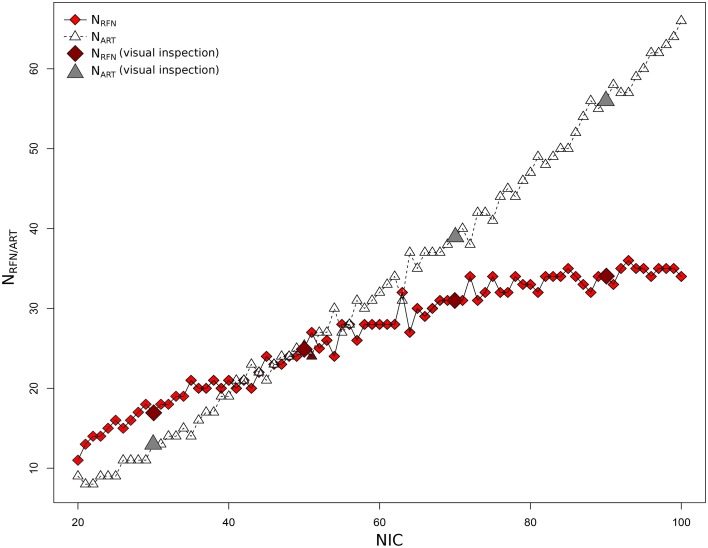
**Number of RFNs and artifact components determined from automated and manual classifications as a function of NICfor dataset 1**.

**Figure 5:**
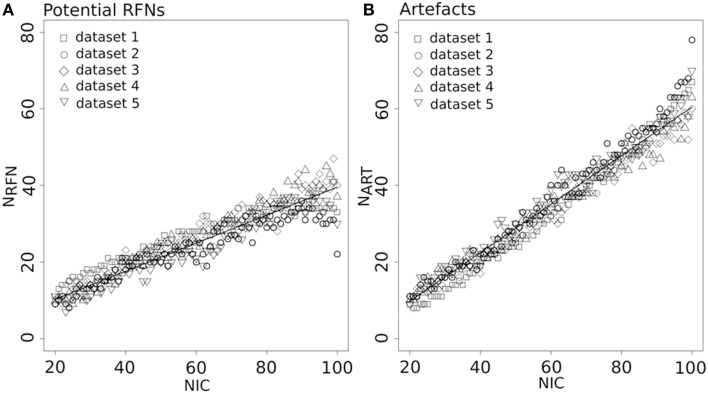
**Number of RFNs (A) and artifact components (B) determined from automated classification as a function of NIC for the datasets 1–5**. The lines indicate the least-square fittings of a linear function to the average data.

Figure 4 depicts the extracted N_RFN_ and N_ART_ for the dataset 1 as a function of NIC. The results from both the automated (with FOCIS) and manual classifications were shown. It is apparent that both N_RFN_ and N_ART_ increase with NIC, but the N_ART_ grows at faster rate than N_RFN_, particularlyat higher NIC. This is further demonstrated by the classification results for the datasets 1–5 summarized in Figure 5. For the group ICA results N_RFN_ and N_ART_ are overall linearly dependent on NIC and the linear correlation coefficient is *R* = 0.93. As summarized in Table 7, the average ratios for N_RFN_/NIC and N_ART_/NICare 0.35 and 0.65, respectively. On average, the number of artifact components is about twice of that for RFNs.

**Table 7:** **The linear least-square fitting results for N_RFN_ and N_ART_ as a function of NIC**.

**Dataset**	**RFN**			**ART**		
	**Ratio**	**Correlation**	**Adjusted *R*^2^**	**Ratio**	**Correlation**	**Adjusted *R*^2^**
1	0.28	0.96	0.93	0.72	0.99	0.99
2	0.29	0.93	0.87	0.71	0.99	0.98
3	0.40	0.98	0.95	0.60	0.99	0.98
4	0.41	0.98	0.96	0.59	0.99	0.98
5	0.36	0.97	0.93	0.64	0.99	0.98
Mean	0.35	0.96	0.93	0.65	0.99	0.98

**Figure 6:**
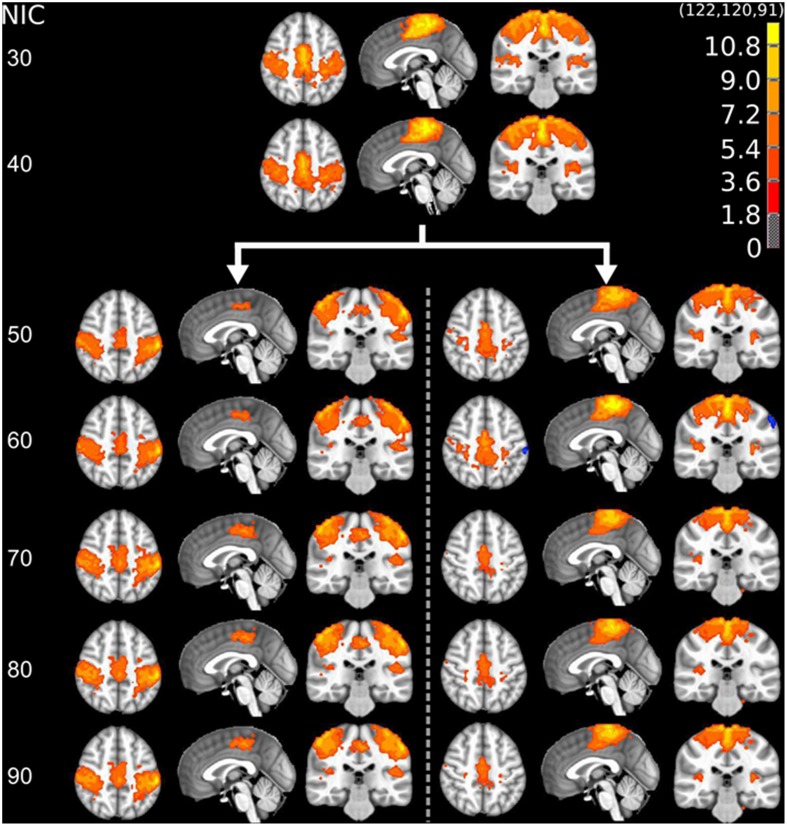
**The tracking results of dataset 1 for a typical RFN through similarity matching**. The motor-sensory functional network was tracked at NIC = 20–100. The IC splits into two potential RFNs with high similarity to the original IC, when NIC = 50.

**Figure 7:**
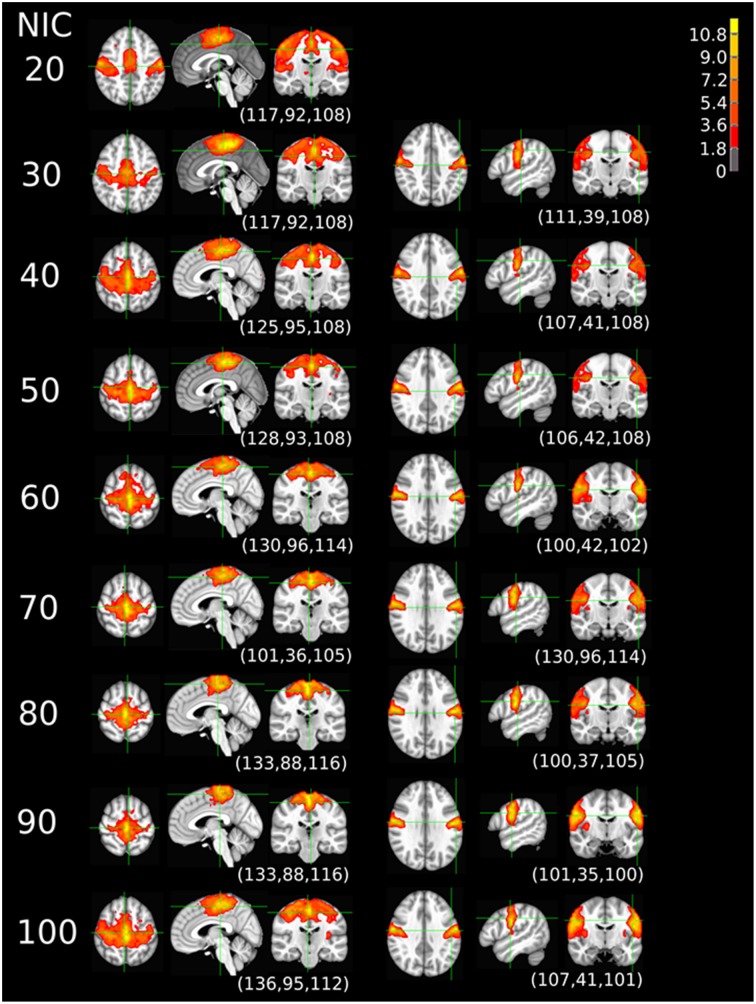
**The tracking results of dataset 4 for a typical RFN through similarity matching**. The motor-sensory functional network was tracked at NIC = 20–100. The IC was split into two potential RFNs with high similarity to the original IC, when NIC = 30.

**Figure 8:**
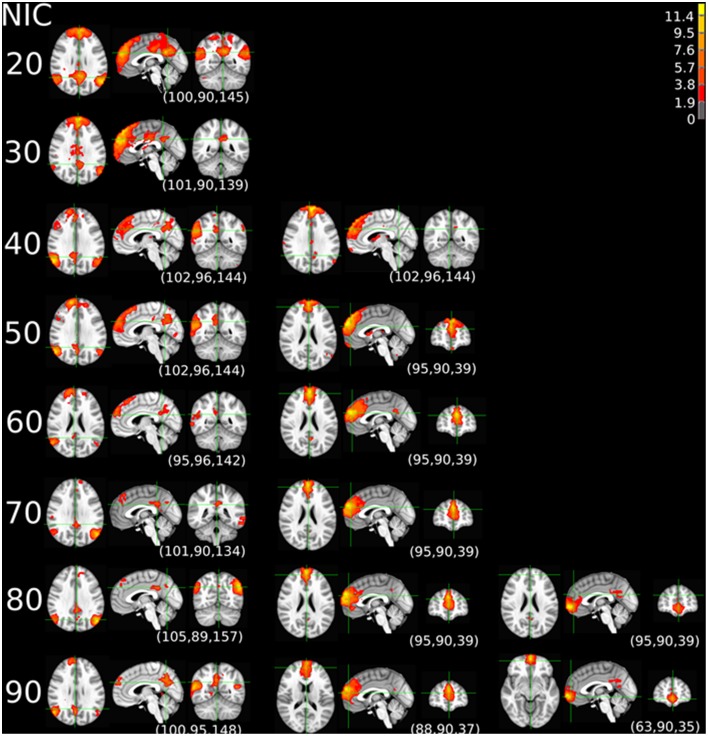
**The tracking results of dataset 4 for a typical RFN through similarity matching**. The primary visual functional network was tracked at NIC = 20–90. The IC did not split in the entire investigated NIC range.

**Figure 9:**
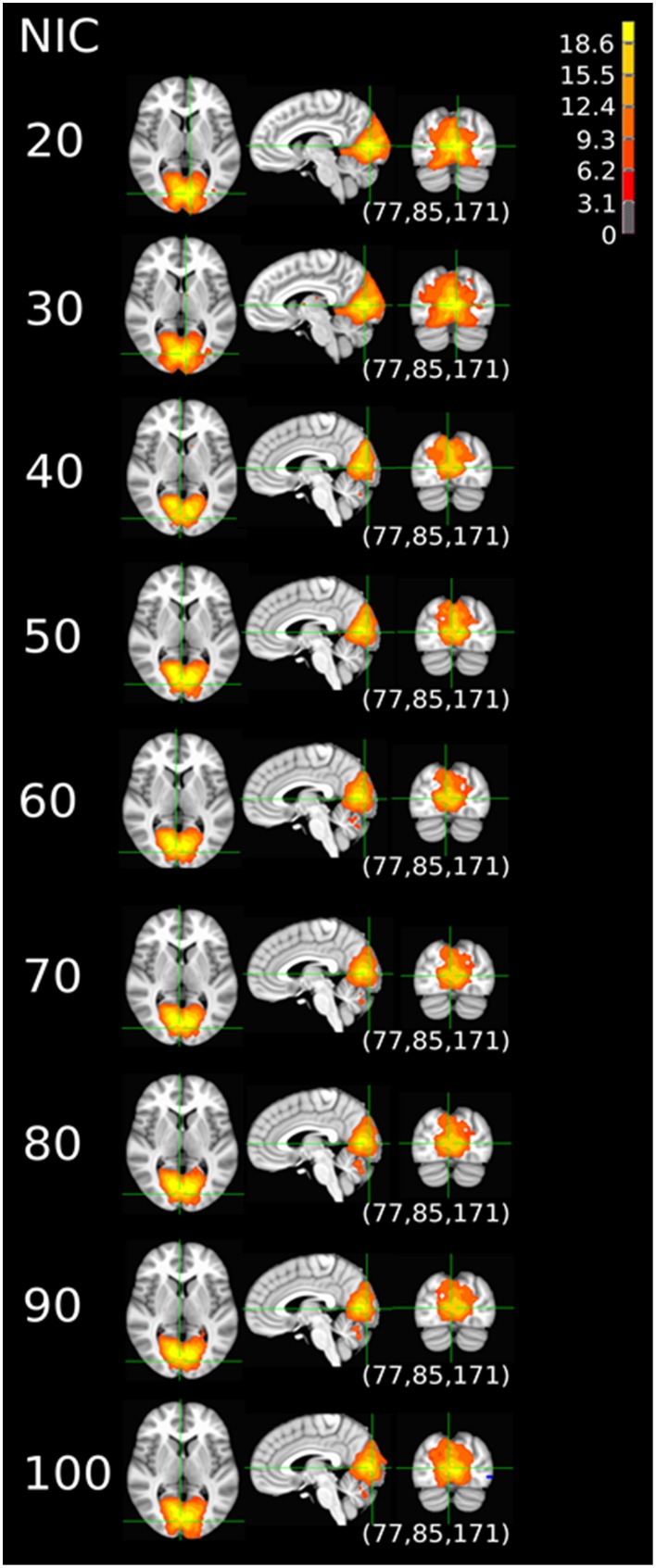
**The tracking results of dataset 4 for a typical RFN through similarity matching**. The DMN was tracked at NIC = 20–100. An anterior and inferior split-offs were detected at NIC = 40 and 80, respectively.

The tracking results of dataset 1 as a function of NIC for the motor-sensory are shown in Figure 6. For this dataset the motor-sensory RFN splits into two ICs starting at NIC = 50. With increasing NIC, the split ICs exhibit consistently high degrees of similarity with the original IC at lower NICs as indicated by the relatively small variations in the rank of matching with the original IC (Table 8). The tracking results of dataset 4 as a function of NIC for the motor-sensory, visual and DMN RFNs are depicted in Figures 7–9, respectively. Similar to the tracking results of dataset 1, the motor-sensory RFN was only split once at NIC = 30 and the split-off ICs display little alterations over the entire investigated NIC interval 20–100. The visual RFN (medial) showed very little change over the entire range of NIC from 20 to 100, indicating that the primary visual system has very strong intra-network association. The DMN was first split into an anterior sub-network at NIC = 40. With further increase in NIC, an inferior split-off was detected at NIC = 80.

**Table 8:** **The ranking and probability of the split ICs for the motor-cortex functional network as a function of NIC**.

**NIC**	**Original IC**		**Split IC**	
	**Ranking**	**Probability**	**Ranking**	**Probability**
30	1	0.98		
40	1	0.89		
50	1	0.56	7	0.10
60	3	0.32	10	0.10
70	2	0.32	3	0.24
80	1	0.35	2	0.23
90	1	0.42	11	0.17

**Figure 10:**
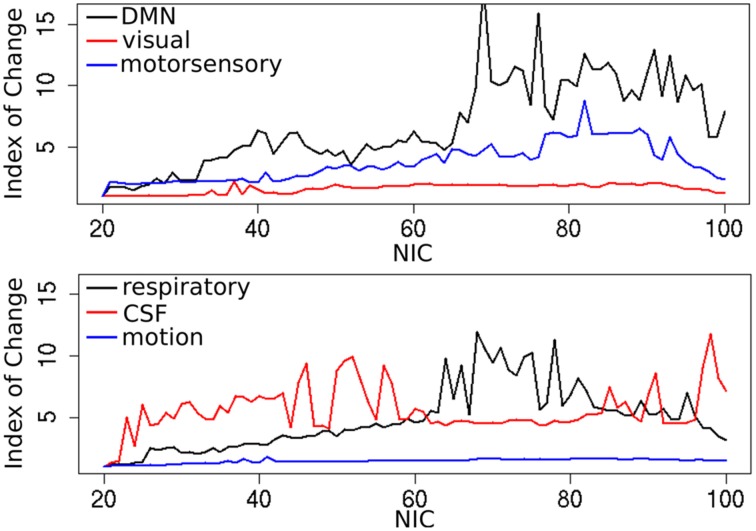
**Change index of spatial overlap and temporal association (CISOTA) as function of NIC for three typical RFNs (top) and artifact (bottom) components**.

**Figure 11:**
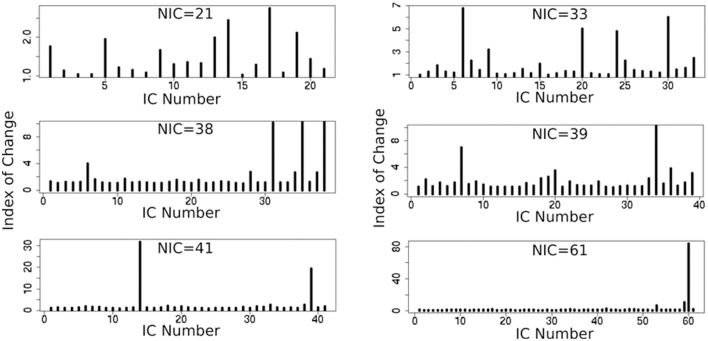
**Cross-sectional plots of change index of spatial overlap and temporal association (CISOTA) for all ICs at different 6 NICs**.

**Figure 12:**
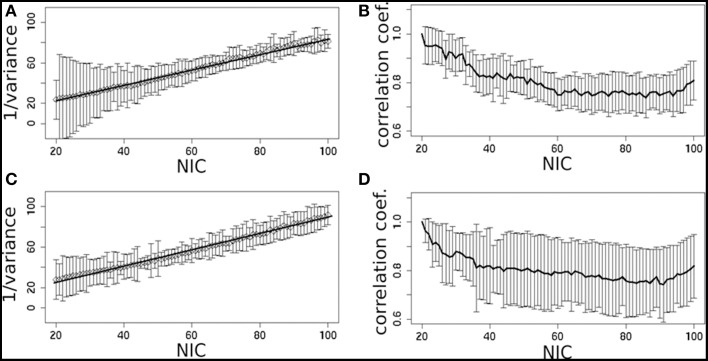
**The effect of NIC on the variance contribution of potential RFNs (A) and artifact components (C)**. The effect of NIC on the IC time courses for potential RFNs **(B)** and artifact components **(D)**.

In addition to the qualitative change associated with the split of a RFN at some specific NICs, with the increase of NIC there exist also gradual and quantitative changes in a given RFN and the trend of change can be quite specific for a given IC. This is clearly illustrated by Figure 10 showing CISOTA as function of NIC for 3 RFNs and artifacts. Irrespective to the categories (RFN or ART), the change of a given IC can be quite steady as for motion artifact component and primary visual network or volatile as for the CSF artifact component and DMN. Figure 11 shows another important aspect of IC change with NIC. At relatively low NIC = 33, incrementing NIC by 1 gives rise to changes in a large number of ICs. When NIC > 40, incrementing NIC by 1 results in changes in a few limited number of ICs. The NIC effect on ICA results can be further appreciated by examining the variance contribution of the ICs and the changes in the associated time courses. As shown Figure 12, the variance contribution from each IC is approximately an inverse function of NICirrespective to whether the IC is a potential RFN or artifact components. As indicated by the Pearson's correlation coefficient of the time courses for a given IC at different NIC (Figures 12B,D), the input NIC produces more significant impact on the time courses ICs when NIC is relatively small (e.g., NIC < 40).

## Discussion

### Classification performance of FOCIS

When the model was trained by using a group ICA dataset and with heavy penalty on false positive rate, FOCIS tends to slightly over-estimate the number of potential RFNs and under-estimate the number of artifact ICs, as indicated by the false negative rate (4/231) in identifying artifact contaminated ICs. Compared to FSL-FIX, the performance of FOCIS in the automated classification of group ICA results is slightly improved in terms of accuracy, precisions and specificity. However, its performance in the classification of single subject data is somewhat poorer with an average accuracy of 91% (Table 5). As reported previously (Griffanti et al., 2014), the true false negative rate for FSL-FIX was 95.1% in the classification of single subject data acquired from 3T scanners with a comparable quality as that for the single subject data studied here. It is noticeable that the previously reported FSL-FIX result was based on training of multiple single-subject datasets, whereas the performance of FOCIS reported here is based on the training of a group ICA dataset (the dataset 1 at NIC = 50). It is probably reasonable to attribute the performance difference to the difference in training.

FSL-FIX employed a large number of features ranging from spatial and temporal characteristics to motion correction and image registration parameters, which is not only useful for identifying rare type of artifacts, but also favorable for situation with large within-class heterogeneity. This may partly explain the good performance of FSL-FIX in the automated classification of single subject ICA results. We cannot generalize that the more features the better performance in classification. In a recent study (Sochat et al., 2014) it was reported that as many as 246 features were employed to achieve a classification accuracy of 87%. Actually, we advocate the selection of features to optimize their classification power, because it does not only help improve the performance of the model but also enhances generalization capability, learning efficiency and model interpretability.

**Figure 13:**
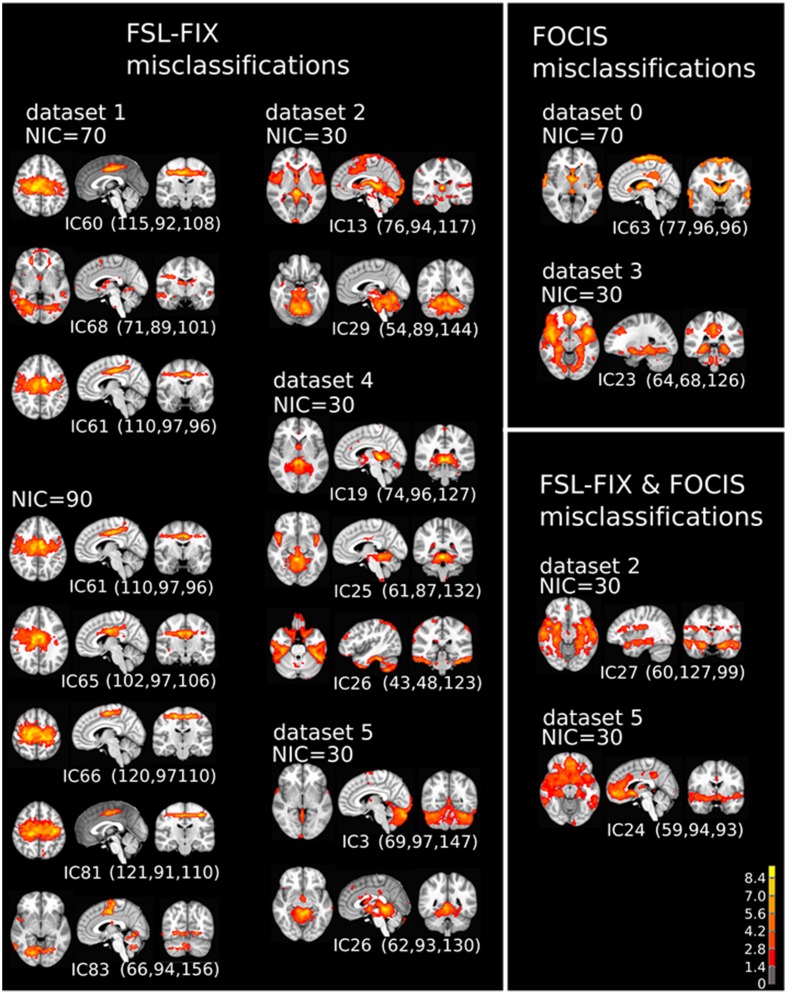
**The spatial patterns of all misclassified ICs summarized in Table 4**.

A closer examination of the misclassified ICs may provide some clues to account for the classification discrepancy between FOCIS and FSL-FIX. The misclassified ICs summarized in Table 4 are depicted in in Figure 13. It is apparent that the main classification discrepancies between FOCIS and FSL-FIX lie in the ICs with relatively simple and regular geometries such as a single plane (e.g., most of ICs in the left column in Figure 13) and ICs having large portion of overlap with the cerebellum, such as most of the ICs in the middle column of Figure 13. As shown in Table 2, one of the five selected features in FOCIS is the ratio of bounding box to voxel account, which is quite sensitive to IC with simple and regular geometries. For simplicity, the tissue templates used in FOCIS does not include cerebellum, it is no surprising that FOCIS reject ICs with large cerebellum overlaps.

It is noteworthy to point out that the using the manually classified ICs from two raters as the ground truth for training is certainly a limitation of the study and can be problematic. However, there is currently no good solution for it unless simulated data are used.

### Classification of borderline ICs

If the results from manual classification are considered as the ground truth, the misclassified ICs by FOCIS belong to the category of false negatives. As shown in the right column of Figure 13, all four ICs misclassified by FOCIS have the characteristics of borderline cases. They may represent meaningful RFN spatial patterns to some degree, but they are contaminated by one or multiple sources of artifacts such as susceptibility, motion, and vascular effects. The human raters classified these ICs as artifact components during the manual inspections due to the strong activities along the most superior location, at the bilateral edges of the temporal lobe, and smaller clusters in white matter and around the contour of the brain, which are classics of motion artifacts. However, the strong activities in the gray matter regions involving posterior cingulate, hypothalamus and thalamus are probably of RFN nature. Therefore, for the “misclassified” ICs, even the visually inspected results may be a matter of dispute.

### The effects of NIC input on the ICA results

As discussed above, ICA can only extract the number of components defined a priori and the RSNs from ICA of resting-state fMRI data are very sensitive to the specified NIC input. This makes it difficult to compare ICA results of resting-state fMRI from different studies. It can even be problematic to compare group differences within the same study. Since the number of ICs are determined both by the potential RFNs and artifacts components, it less likely to find a ground truth common NIC for the different datasets. Even if the same model order parameter was used in the different studies, the obstacle could still remain, because the data acquisition and structured noise may differ substantially.

Different methods (Cordes and Nandy, 2006; Li et al., 2007; Xie et al., 2009; Chen et al., 2010; Beckmann, 2012) have been employed to improve the estimation of dimensionality. However, the rationality of the applied criterion is still questionable, because the estimates are typically dependent on SNR of the data and preprocessing pipeline steps, but not directly related to the neurophysiological properties. The use of a relatively low NIC in the range of 20–30 has the risk to discard potentially useful information, because the more complex RFNs are left aside as mixtures of real independent constituents. On the hand, the use of a large NIC can results in an excessive number of components with dissociated sources. Furthermore, algorithmic variability of ICA decomposition increases with NIC, we are left with a trade-off to choose between high modularity versus reproducibility and over-fitted ICs.

From the experimental point of view, there are nearly proportional increases in both N_RFN_ and N_ART_ with NIC for most of the datasets except for dataset 1 (see Figure 4). Neither the variation of N_RFN_ and N_ART_ as a function of NIC, nor the tracking the variance contribution of the ICs showed any clear sign of reaching a steady state or transition point. For dataset 1 there appears to be a clear upper limit for the number of RFNs at NIC = 70 ± 10 beyond which only the number of artifact ICs increases as one increases NIC (Figure 4). This result is quite consistent with a previous report (Abou Elseoud et al., 2010; Elseoud et al., 2011) on the effect of NIC selection in group ICA. It was reported that NIC = 70 ± 10 offered a more detailed evaluation of RSNs in spatial pattern, whereas NIC > 100 produced a decrease in ICA repeatability, but no gain in either volume or mean z-score results (Abou Elseoud et al., 2010; Elseoud et al., 2011). It is apparent that we cannot draw a general conclusion using the observations from a couple of datasets. It is unlikely that we can find a ground truth common NIC for the different datasets, because the number of ICs are determined both by the potential RFNs and artifacts components. From neuroscientific point of view, it is probably more productive to try to find a set of RFN template common for normal controls through ICA or other data-driven approaches. Therefore, tracking the spatial and temporal changes of some typical RFNs as a function of NIC can be very useful in gaining insight into how the RFNs are affected by selected NIC and deriving criterion for defining dimensionality of ICA in resting-state fMRI.

## Conclusion

We have described a new tool, FOCIS, for the automated classification of artifact components in ICA results of resting-state fMRI data. Based on training of a group ICA dataset, FOCIS achieved on average 98 and 91% classification accuracy on the group and single subject ICA datasets, respectively. Therefore, FOCIS can be a very useful tool for assisting automated classification of ICA results from resting-state fMRI. The classification model employs the five most significant features to catch relevant spatial and temporal characteristics with most discriminative power to differentiate RFNs from artifacts components. Once trained a minimum account of hand-labeled data, FOCIS can be applied for automated classification of both group and single subject ICA results from resting-state fMRI datasets of different acquisition parameters without further intervention.

FOCIS is particularly useful for the studies involving a large number of ICs such as in the study of ICA dimensionality problem of resting-state fMRI datasets. With FOCIS, we were able to conduct systematically group ICA of six resting-state fMRI datasets acquired at different sites using different protocols as a function of NIC varied systematically from 20 to 100. We found that NIC can critically affect both the spatial pattern and temporal characteristics of the RFNs. The dimensionality problem deserves further investigations, because the input NIC can substantially affect group ICA results and the outcome of group comparison studies.

### Conflict of interest statement

The authors declare that the research was conducted in the absence of any commercial or financial relationships that could be construed as a potential conflict of interest.

## Abbreviations

ICA: Independent Component Analysis
NIC: Number of Independent Components
SVM: Support Vector Machines
IC: Independent Component
FOCIS: Feature Optimized Classification of Independent components with SVM
(fsl) FIX: FMRIB's ICA-based X-noiseifier
RFN: Resting-state Functional Networks
CSF: Cerebral Spinal Fluid
MDL: Minimum Description Length
PPCA: Probabilistic Principle Component Analysis
AIC: Akaike Infomormation Criteria
FWHM: Full Width at Half Maximum
DMN: Default-Mode Network
Class RFN: Classification class of Potential Resting-state Functional Networks
Class ART: Classification class of artefacts and otherwise components of non-interest
RBF: Radial Basis Function (kernel)
N_RFN_: Number of members of class RFN
N_ART_: Number of members of class ART
CISOTA: Change Index of Spatial Overlap and Temporal Association.
