# Supplementary material for: Dimensionality of ICA in resting-state fMRI investigated by feature optimized classification of independent components with SVM
Source: Front Hum Neurosci. 2015 May 8;9:259. doi: 10.3389/fnhum.2015.00259 (PMC4424860; doi:10.3389/fnhum.2015.00259)
Supplement: Supplementary file 1 [file DataSheet1.DOCX]

**Appendix A: Features**

## Measures based on spatial distribution of IC maps

Several measures were devised to characterize the spatial distribution of IC map values. For a given distribution, the *kurtosis* is a measure of the sparseness and the *skewness* is a measure of asymmetry ([Formisano et al., 2002](#_ENREF_22);[Suzuki et al., 2002](#_ENREF_42)). They are both zero for Gaussian distributions. In this study, the features were calculated for a given IC map according to the following:

$$kurtosis=\frac{\sum_{k=1}^{N} z_{k}^{4}}{N}-3$$

$$skewness=\frac{\sum_{k=1}^{N} z_{k}^{3}}{N}$$

where *z_k_* is the value of the *k-*th voxel and *N* is the total number of voxels.

*Spatial entropy* measures information content of the IC map value distribution. It is expected to be higher for ICs with widely distributed values. The spatial entropy *H_sp_* is calculated according to the following:

$$H_{sp}=\sum_{y=1}^{N_{y}} hist(y)\log_{2}(hist(y))$$

Where *hist* is the voxel values summed over *N_y_* number of bins ([De Martino et al., 2007](#_ENREF_19)).

## Measures derived from the spatial structure of IC maps

Several features were included to characterize the spatial structure of meaningful ICs. *Positive t-value with grey-matter overlap* (*gm_+_*) and *negative t-value with grey-matter overlap* (*gm_-_*) quantifies the characteristic that meaningful ICs should exhibit activations in cortical grey-matter. The features were calculated according to the following:

$$gm_{+/-}=\frac{\sum_{v} (t_{+/-}(v)w(v))}{\sum_{v} t_{+/-}(v)}$$

Summed over all voxels *v*, *gm_+_*$\mathrm{gm}_{+}$ is the ratio of cortical grey-matter contribution from an IC’s positive values and *gm_-_*$\mathrm{gm}_{-}$ is the corresponding feature for negative values. *t_+/-_(v)* is the IC’s spatial map value at voxel *v* after the following threshold:

$$t_{+}(v)=\left\{\begin{matrix} \begin{matrix} t(v) & if & t(v)\geq0 \end{matrix} \\ \begin{matrix} 0 & otherwise \end{matrix} \end{matrix}\right.$$

$$t_{-}(v)=\left\{\begin{matrix} \begin{matrix} -t(v) & if & t(v)\leq0 \end{matrix} \\ \begin{matrix} 0 & otherwise \end{matrix} \end{matrix}\right.$$

and *w(v)* is the cortical grey-matter mask. Both the IC spatial map and cortical grey-matter mask were thresholded at p<0.05 with a binary operation before calculations.

A number of features were devised to characterize an IC spatial map through its clusters. The coordinates of a cluster’s center of mass and peak activation voxel were obtained using the nearest-neighbor cluster detection algorithm in the AFNI program *3dclust.* The feature *center of mass of clusters within cluster* characterizes a cluster’s shape by checking of if its center of mass lies within the cluster itself. The feature *peak voxel of clusters within grey-matter* characterizes a cluster’s location by checking if the cluster’s peak activation voxel resides within the binary cortical grey-matter mask*.* If the nearest neighbor clustering algorithm returns more than one cluster for a given IC, the aforementioned features will be calculated individually and a weighted average of the individual clusters’ sizes will be used as the feature value for the IC.

The following features were devised specifically for the purpose of characterizing motion artifacts typically observed as rings around the brain or between the two hemispheres. Let *BB_AP_*, *BB_SP_*, and *BB_LR_* denote a cluster’s bounding-box size in the anterior-posterior, superior-inferior, and right-right directions respectively. The feature *bounding-box irregularity* is then be defined as:

$$Boundingbox.Irreg=\frac{\min(BB_{AP},BB_{SI},BB_{LR})}{\max(BB_{AP},BB_{SI},BB_{LR})}$$

The feature *A-P irregularity of the clusters’ Bounding-box* (*Boundingbox.Irreg.AP*) is a similar measure to *Boundingbox.Irreg*, but is tailored specifically for motion artifacts between the two hemispheres and defined as:

$$Boundingbox.Irreg.AP=\frac{\min(BB_{SI},BB_{LR})}{BB_{AP}}$$

The feature *cluster bounding-box to voxel count ratio* exploits the fact that ring shaped clusters from motion artifacts is relatively empty on the inside of the ring. It is defined as the ratio of a cluster’s size in voxels over the cluster’s bounding-box volume.

## Features derived from time-course and frequency spectrum

Two sets of features characterizing the temporal structure and spectral decomposition were devised based on two types of time-courses:

1. *fMRI time-course*: The ideals are defined as the average time-course over all voxels inside a thresholded (p<0.05) IC spatial map. For a given IC, all ideals (one for each subject in a dataset) were averaged together, resulting in a single time-course for a given IC.
2. *IC time*-course: The concatenated IC time-course.

Besides the types of time-course used, the two sets of features are otherwise identical.

The feature *1-lag autocorrelation* of a time-course is a measure of temporal structure. BOLD responses is expected to have high values of autocorrelation while white noise conversely is characterized by an autocorrelation function that is one at zero lag and zero everywhere else. For each IC, one-lag autocorrelation was estimated as:

$$r=\frac{\frac{1}{T-1}\sum_{t=1}^{T-1} a(t)a(t+1)}{\frac{1}{T}\sum_{t=1}^{T} a(t)^{2}}$$

where *a(t)* is the value of the time course at time *t* and T is the number of time points.

*Entropy of IC time course* is a measure of information content of the time-course. White noise is expected to have higher temporal entropy than periodic signals of BOLD responses. It is estimated as:

$$H_{t}=\sum_{y=1}^{N_{y}} hist_{t}(y)\log_{2}(hist_{t}(y))$$

where *hist_t_(y)* is the time course values histogram computed over *N_y_* number of bins.

The average power spectrum density (PSD) was calculated for each time-course using the fast Fourier transform method.

*Dynamic range* of a time course is defined as maximum of the PSD minus the mean of the high frequencies (0.03 < f < 0.05 Hz).

*Frequency ratio* of a time-course is the defined as the ratio of the area the PSD curve for the low frequencies (0.00 < f < 0.02 Hz) over the high frequencies (0.03 < f < 0.05 Hz).

**Appendix B: Code snippets for the feature calculation**

#============================

# Spatial mask-based features

#============================

#!/bin/bash

components**=`ls <name_of_components>`**

**for** c **in** **$components**

**do**

3dcalc -a ''**$c**'' **-**expr 'ispositive(a)*a' **-**prefix pv_**$c**

3dcalc -a ''**$c**'' **-**expr 'isnegativ(a)*-a' **-**prefix nv_**$c**

3dmaskdump **-**mask **<**path_to_brain_mask**>** **-**noijk -o Dpref_**$c** pv_**$c**

3dmaskdump **-**mask **<**path_to_brain_mask**>** **-**noijk -o Dnref_**$c** nv_**$c**

3dmaskdump **-**mask **<**path_to_brain_mask**>** **-**noijk -o td **$c**

thresholds**=`thrhld.R td`**

# Contents of thrhld.R

# -----------------------------------------------------------------------

#!/usr/bin/Rscript

args**<-**commandArgs**(**TRUE**);**

dump**<-**read.table**(**args**[**1**]);**

data**<-**dump**[**dump**!=**0**];**

**print(** c**(**mean**(**data**)+**2*****sd**(**data**),-**1***(**mean**(**data**)-**2*****sd**(**data**)))** **);**

# -----------------------------------------------------------------------

IFS**=**" "**;** **set** **--** **$thresholds;** pt**=$2;** nt**=$3;** **rm** td

3dcalc -a ''pv_**$c**'' **-**expr "ispositive(a-$pt)" **-**prefix pthr_**$c**

3dcalc -a ''nv_**$c**'' **-**expr "ispositive(a-$nt)" **-**prefix nthr_**$c**

3dcalc -a '<path to cortical grey-matter mask>' -b ''pthr_**$c**'' **-**expr 'step(a)*step(b)' **-**prefix pRes_**$c**

3dcalc -a '<path to cortical grey-matter mask>' -b ''nthr_**$c**'' **-**expr 'step(a)*step(b)' **-**prefix nRes_**$c**

3dmaskdump **-**mask **<**path_to_brain_mask**>** **-**noijk -o Dpos_**$c** pRes_**$c**

3dmaskdump **-**mask **<**path_to_brain_mask**>** **-**noijk -o Dneg_**$c** nRes_**$c**

**done**

mskfeat.R

# Contents of mskfeat.R

# -----------------------------------------------------------------------

#!/usr/bin/Rscript

res.pos **<-** list.files**(**pattern**=**'Dpos_*'**);**

res.neg **<-** list.files**(**pattern**=**'Dneg_*'**);**

ref.pos **<-** list.files**(**pattern**=**'Dpref_*'**);**

ref.neg **<-** list.files**(**pattern**=**'Dnref_*'**);**

N **<-** length**(**res.pos**);**

outp **<-** array**(** 0 **,** dim**=**c**(**N**,**2**)** **);**

**for** **(**i in 1**:**N**)** **{**

p.res **<-** read.table**(**res.pos**[**i**]);**

n.res **<-** read.table**(**res.neg**[**i**]);**

p.ref **<-** read.table**(**ref.pos**[**i**]);**

n.ref **<-** read.table**(**ref.neg**[**i**]);**

Tp **<-** sum**(**p.ref**[,**1**]);**

Tn **<-** sum**(**n.ref**[,**1**]);**

pos.con **<-** sum**(**p.res**[,**1**])/**Tp**;**

neg.con **<-** sum**(**n.res**[,**1**])/**Tn**;**

outp**[**i**,**1**]** **<-** pos.con**;**

outp**[**i**,**2**]** **<-** neg.con**;**

**}**

write.table**(**results**,** file**=**'mask_feat'**);**

# -----------------------------------------------------------------------

#=============================

# Average time-course features

#=============================

#!/bin/bash

components**=`ls <name_of_components>`**

**touch** tc_feat

**for** c **in** **$components**

**do**

3dcalc -a ''**$c**'' **-**expr 'ispositive(a)' **-**prefix pm_**$c**

3dcalc -a ''**$c**'' **-**expr 'isnegative(a)' **-**prefix nm_**$c**

3dcalc -a ''**$c**'' -b ''pm_**$c**'' **-**expr 'a*b' **-**prefix pv_**$c**

3dcalc -a ''**$c**'' -b ''nm_**$c**'' **-**expr '-a*b' **-**prefix nv_**$c**

3dmaskdump **-**mask **<**path_to_brain_mask**>** **-**noijk -o td **$c**

thresholds**=`thrhld.R td`**

# Contents of thrhld.R

# -----------------------------------------------------------------------

#!/usr/bin/Rscript

args**<-**commandArgs**(**TRUE**);**

dump**<-**read.table**(**args**[**1**]);**

data**<-**dump**[**dump**!=**0**];**

**print(** c**(**mean**(**data**)+**2*****sd**(**data**),-**1***(**mean**(**data**)-**2*****sd**(**data**)))** **);**

# -----------------------------------------------------------------------

OLD_IFS**=$IFS;** IFS**=**" "**;** **set** **--** **$thresholds;** pt**=$2;** nt**=$3;** **rm** td

3dcalc -a ''pv_**$c** '' **-**expr "ispositive(a-$pt)" **-**prefix pthr_**$c**

3dcalc -a ''nv_**$c**'' **-**expr "ispositive(a-$nt)" **-**prefix nthr_**$c**

3dcalc -a ''pthr_**$c**'' -b ''nthr_**$c**'' **-**expr 'a+b' **-**prefix component_mask.nii

IFS**=$OLD_IFS**

data**=`ls preproc_*.nii`**

**for** dat **in** **$data**

**do**

3dROIstats **-**mask component_mask.nii **$dat** **>** ideal

calc_tc.R ideal **>>** TMP_tc_feat_**$c**

# Contents of calc_tc.R

# -----------------------------------------------------------------------

#!/usr/bin/Rscript

require**(**entropy**);**

require**(**stats**);**

require**(**pracma**);**

calcPSD **<-** function**(**tc**,** norm**=**TRUE**,** tavg**=**0**,** psdavg**=**0 **)** **{**

sig **<-** tc**;**

N **<-** length**(**tc**);**

**if(**tavg **>** 0**)** **{**

sig **<-** rollingAveraget**(**tc**,** tavg**);**

**}**

fourier **<-** fft**(** tc **);**

power **<-** **(** Mod**(**fourier**)** **)^**2**;**

power_fh **<-** power**[**1**:(**N**/**2**)];**

**if** **(**normalize**==**TRUE**)** **{**

T **<-** sqrt**(sum(**td**^**2**))***length**(**tc**);**

power_fh **<-** power_fh**/**T**;**

**}**

**if** **(**psdavg **>** 0**)** **{**

power_fh **<-**rollingAverage**(**power_fh**,** psdavg**);**

**}**

xaxis **<-** 1**:(**N**/**2**)/**N**;**

ans **<-** array**(** 0**,** dim**=**c**(**2**,**N**/**2**)** **);**

ans**[**1**,]** **<-** xaxis**;**

ans**[**2**,]** **<-** power_fh**;**

**return(**ans**);**

**}**

args **<-** commandArgs**(**TRUE**);**

inputData **<-** args**[**1**];**

data **<-** read.table**(**inputData**);**

data **<-** as.double**(**array**(**data**[-**1**,**3**]));**

cor **<-** acf**(**data**,** type**=**"correlation"**,** lag.max**=**1**,** plot**=**FALSE**);**

one.lag **<-** cor**$acf[**2**];**

W **<-** 2 ***** IQR**(**data**)** ***** length**(**data**)^(-**1**/**3**);**

nrBins **<-** ceiling**(** **(**range**(**data**)[**2**]** **-** range**(**data**)[**1**]** **)** **/** W **)**

entrop **<-** entropy**(**discretize**(**data**,**nrBins**));**

psd **<-** calcPSD**(**data**);**

sec **<-** floor**(**length**(**psd**[**1**,])/**10**)**

LF **<-** list**(**"x"**=**psd**[**1**,**1**:(**sec*****2**)],** "y"**=**psd**[**2**,**1**:(**sec*****2**)]);**

HF **<-** list**(**"x"**=**psd**[**1**,(**sec*****3**):(**sec*****5**)],** "y"**=**psd**[**2**,(**sec*****3**):(**sec*****5**)]);**

dynamic.range **<-** max**(**LF**$y)-**mean**(**HF**$y);**

AUC.LF **<-** trapz**(**LF**$x,**LF**$y);**

AUC.HF **<-** trapz**(**HF**$x,**HF**$y);**

ratio **<-** AUC.LF**/**AUC.HF**;**

**print(** c**(**one.lag**,** entrop**,** dynamic.range**,** ratio**)** **);**

# -----------------------------------------------------------------------

**done**

avg_tc.R TMP_tc_feat_**$c** **>>** tc_feat

# Contents of avg_tc.R

# -----------------------------------------------------------------------

#!/usr/bin/Rscript

args**<-**commandArgs**(**TRUE**);**

data**<-**read.table**(**args**[**1**]);**

one.lag **<-** mean**(**data**[,**2**]);**

entrop **<-** mean**(**data**[,**3**]);**

dynamic.range **<-** mean**(**data**[,**4**]);**

ratio **<-** mean**(**data**[,**5**]);**

**print(** c**(**one.lag**,** entrop**,** dynamic.range**,** ratio**)** **);**

# -----------------------------------------------------------------------

**<**clean up code...**>**

**done**

#===============================

# Spatial t-map Cluster features

#===============================

#!/bin/bash

components**=`ls <name_of_components>`**

**touch** clust_feat

**for** c **in** **$components**

**do**

3dmaskdump **-**mask **<**path_to_brain_mask**>** **-**noijk -o df **$c**

thresholds**=`thrhld.R td`**

# Contents of thrhld.R

# -----------------------------------------------------------------------

#!/usr/bin/Rscript

args**<-**commandArgs**(**TRUE**);**

dump**<-**read.table**(**args**[**1**]);**

data**<-**dump**[**dump**!=**0**];**

**print(** c**(**mean**(**data**)+**2*****sd**(**data**),-**1***(**mean**(**data**)-**2*****sd**(**data**)))** **);**

# -----------------------------------------------------------------------

IFS**=**" "**;** **set** **--** **$thresholds;** pt**=$2;** nt**=$3;** **rm** td

3dclust **-**savemask clusterMask.nii **-**dxyz**=**1 **-**1abs **-**1clip **$pt** 2 20 **$c** **>** clres

**sed** '/#.*$/d' clres **>** cl_results

**rm** clres

extInfo.R cl_results

# Contents of extInfo.R

# -----------------------------------------------------------------------

#!/usr/bin/Rscript

args **<-** commandArgs**(**TRUE**);**

clusterDump **<-** tryCatch**({**

read.table**(**args**[**1**]);**

**},** error **=** function **(**war**)** **{**

**print(** c**(**0**,**0**,**0**)** **);**

q**(**"no"**);**

**});**

clusterSizes **<-** clusterDump**[,**1**];**

nrOfClusters **<-** length**(**clusterSizes**);**

totalClusterSize **<-** sum**(**clusterSizes**);**

a.weights **<-** array**(**0**,** dim**=**nrOfClusters**);**

a.coords **<-** array**(**0**,** dim**=**c**(**nrOfClusters**,**6**)** **);**

a.BBparams **<-** array**(**0**,** dim**=**c**(**nrOfClusters**,**3**));**

**for** **(** i in 1**:**nrOfClusters **)** **{**

a.coords**[**i**,**1**]** **<-** clusterDump**[**i**,**2**];**

a.coords**[**i**,**2**]** **<-** clusterDump**[**i**,**3**];**

a.coords**[**i**,**3**]** **<-** clusterDump**[**i**,**4**];**

a.coords**[**i**,**4**]** **<-** clusterDump**[**i**,**14**];**

a.coords**[**i**,**5**]** **<-** clusterDump**[**i**,**15**];**

a.coords**[**i**,**6**]** **<-** clusterDump**[**i**,**16**];**

a.weights**[**i**]** **<-** clusterSizes**[**i**]** **/** totalClusterSize**;**

min.RL **<-** clusterDump**[**i**,**5**];** max.RL **<-** clusterDump**[**i**,**6**];**

min.AP **<-** clusterDump**[**i**,**7**];** max.AP **<-** clusterDump**[**i**,**8**];**

min.IS **<-** clusterDump**[**i**,**9**];** max.IS **<-** clusterDump**[**i**,**10**];**

cluster.vol **<-** clusterDump**[**i**,**1**];**

RL **<-** max.RL **-** min.RL**;**

AP **<-** max.AP **-** min.AP**;**

IS **<-** max.IS **-** min.IS**;**

BB.vol **<-** RL*****AP*****IS**;**

BB.max.irreg **<-** **(**max**(**RL**,**AP**,**IS**)-**min**(**RL**,**AP**,**IS**))/**max**(**RL**,**AP**,**IS**);**

BB.midline.irreg **<-** AP**/**RL**;**

BB.fill **<-** cluster.vol**/**BB.vol**;**

a.BBparams**[**i**,**1**]** **<-** BB.max.irreg**;**

a.BBparams**[**i**,**2**]** **<-** BB.midline.irreg**;**

a.BBparams**[**i**,**3**]** **<-** BB.fill**;**

**}**

write.table**(**a.coords**,** file**=**"extInfo_coords"**);**

write.table**(**a.weights**,** file**=**"extInfo_weights"**);**

write.table**(**a.BBparams**,** file**=**"extInfo_BBparams"**);**

# -----------------------------------------------------------------------

**if** **[** **-f** 2nd_extractInfo_weights **];**

**then**

**sed** 's/".*"//g' extInfo_weights **>** weights

**sed** 's/".*"//g' extInfo_coords **>** coords

**sed** 's/".*"//g' extInfo_BBparams **>** BBparams

**rm** cl_results

**rm** extInfo*****

counter**=**1**;**

**while** **read** line

**do**

**if** **[[** **!** **$line** **=~** **[^[:**space**:]]** **]]** **;** **then**

**continue**

**fi**

**set** **--** **$line**

CMx**=$1**

CMy**=$2**

CMz**=$3**

PKx**=$4**

PKy**=$5**

PKz**=$6**

3dcalc -a **$c** **-**expr "((1/2)-(1/52))*step(4-(x-$CMx)*(x-$CMx)-(y-$CMy)*(y-$CMy)-(z-$CMz)*(z-$CMz))" **-**prefix CMc.nii

3dcalc -a **$c** **-**expr "(1/52)*step(40-(x-$CMx)*(x-$CMx)-(y-$CMy)*(y-$CMy)-(z-$CMz)*(z-$CMz))" **-**prefix CMs.nii

3dcalc -a CMc.nii -b CMs.nii **-**expr "a+b" **-**prefix CMpCl.nii

**rm** CMc.nii CMs.nii

3dcalc -a **$c** **-**expr "((1/2)-(1/52))*step(4-(x-$PKx)*(x-$PKx)-(y-$PKy)*(y-$PKy)-(z-$PKz)*(z-$PKz))" **-**prefix PKc.nii

3dcalc -a **$c** **-**expr "((1/52))*step(4-(x-$PKx)*(x-$PKx)-(y-$PKy)*(y-$PKy)-(z-$PKz)*(z-$PKz))" **-**prefix PKs.nii

3dcalc -a PKc.nii -b PKs.nii **-**expr "a+b" **-**prefix PKpCl.nii

**rm** PKc.nii PKs.nii

3dcalc -a '<path to cortical grey-matter mask>' -b 'PKpCl' **-**expr 'b*a' **-**prefix PK_clInfo.nii

3dcalc -a 'clusterMask.nii' -b 'CMpCl' **-**expr "b*within(a,$counter,$counter)" **-**prefix CM_clInfo.nii

3dmaskdump **-**mask **<**path_to_brain_mask**>** **-**noijk -o PK_clInfo_**$counter** PK_clInfo.nii

3dmaskdump **-**mask **<**path_to_brain_mask**>** **-**noijk -o CM_clInfo_**$counter** CM_clInfo.nii

counter**=$(($counter+**1**))**

**rm** *****pCl.nii

**rm** *****_clusterInfo.nii

**done** **<** coords

sumClust.R **>>** clust_feat

# Contents of sumClust.R

# -----------------------------------------------------------------------

#!/usr/bin/env Rscript

CM.dumps **<-** list.files**(**pattern**=**"CM_clInfo_*"**);**

PEAK.dumps **<-** list.files**(**pattern**=**"PK_clInfo_*"**);**

weights.dump **<-** read.table**(**'weights'**);**

BBparams.dump **<-** read.table**(**'BBparams'**);**

cluster.weights **<-** weights.dump**[,**1**];**

n **<-** length**(**CM.dumps**);**

m **<-** length**(**cluster.weights**);**

r.o **<-** array**(** 0 **,** dim**=**c**(**n**,** 5**)** **);**

**for** **(**i in 1**:**n**)** **{**

CM.file **<-** read.table**(** CM.dumps**[**i**]** **);**

CM.data **<-** CM.file**[,**1**];**

CM.data **<-** CM.data**[**CM.data **!=** 0**];**

PEAK.file **<-** read.table**(** PEAK.dumps**[**i**]** **);**

PEAK.data **<-** PEAK.file**[,**1**];**

PEAK.data **<-** PEAK.data**[**PEAK.data **!=** 0**];**

r.o**[**i**,**1**]** **<-** sum**(**CM.data**)** ***** cluster.weights**[**i**];**

r.o**[**i**,**2**]** **<-** sum**(**PEAK.data**)** ***** cluster.weights**[**i**];**

r.o**[**i**,**3**]** **<-** BBparams.dump**[**i**,**1**]** ***** cluster.weights**[**i**];**

r.o**[**i**,**4**]** **<-** BBparams.dump**[**i**,**2**]** ***** cluster.weights**[**i**];**

r.o**[**i**,**5**]** **<-** BBparams.dump**[**i**,**3**]** ***** cluster.weights**[**i**];**

**}**

outp **<-** colSums**(**results**);**

**print(** outp **);**

# -----------------------------------------------------------------------

**<**clean up code...**>**

**else**

zeroFill.R **>>** clust_feat

# Contents of zeroFill.R

# -----------------------------------------------------------------------

#!/usr/bin/env Rscript

**print(** c**(**0**,**0**,**0**,**0**,**0**)** **)**

# -----------------------------------------------------------------------

**fi**

**done**

# ===================================

# Spatial t-map density plot features

# ===================================

#!/bin/bash

components**=`ls <name_of_components>`**

**touch** hist_feat

**for** c **in** **$components**

**do**

3dmaskdump **-**mask **<**path_to_brain_mask**>** **-**noijk -o D_**$c** **$c**

calcHistFeat.R D_**$c** **>>** hist_feat

# Contents of calcHistFeat.R

# -----------------------------------------------------------------------

#!/usr/bin/Rscript

require**(**entropy**);**

require**(**e1071**);**

args **<-** commandArgs**(**TRUE**);**

inputData **<-** args**[**1**];**

data **<-** read.table**(**inputData**);**

dens **<-** density**(**data**[,**1**]);**

kurt **<-** kurtosis**(**dens**$y);**

skew **<-** skewness**(**dens**$y);**

spat.entropy **<-** entropy**(**dens**$y);**

**print(** c**(**kurt**,** skew**,** spat.entropy**)** **);**

# -----------------------------------------------------------------------

**rm** D_**$c**

**done**

#========================

# IC time-course features

#========================

#!/usr/bin/Rscript

require**(**entropy**);**

require**(**stats**);**

require**(**pracma**);**

calcPSD **<-** function**(**tc**,** norm**=**TRUE**,** tavg**=**0**,** psdavg**=**0 **)** **{**

sig **<-** tc**;**

N **<-** length**(**tc**);**

**if(**tavg **>** 0**)** **{**

sig **<-** rollingAveraget**(**tc**,** tavg**);**

**}**

fourier **<-** fft**(** tc **);**

power **<-** **(** Mod**(**fourier**)** **)^**2**;**

power_fh **<-** power**[**1**:(**N**/**2**)];**

**if** **(**normalize**==**TRUE**)** **{**

T **<-** sqrt**(sum(**td**^**2**))***length**(**tc**);**

power_fh **<-** power_fh**/**T**;**

**}**

**if** **(**psdavg **>** 0**)** **{**

power_fh **<-**rollingAverage**(**power_fh**,** psdavg**);**

**}**

xaxis **<-** 1**:(**N**/**2**)/**N**;**

ans **<-** array**(** 0**,** dim**=**c**(**2**,**N**/**2**)** **);**

ans**[**1**,]** **<-** xaxis**;**

ans**[**2**,]** **<-** power_fh**;**

**return(**ans**);**

**}**

all.data **<-** read.table**(**'melodic_mix'**);**

nrComponents **<-** dim**(**alldata**)[**2**];**

output **<-** array**(**0**,** dim**=**c**(**nrComponents**,**4**));**

**for** **(** i in 1**:**nrComponents **)** **{**

data **<-** all.data**[,**i**];**

cor **<-** acf**(**data**,** type**=**"correlation"**,** lag.max**=**1**,** plot**=**FALSE**);**

one.lag **<-** cor**$acf[**2**];**

W **<-** 2 ***** IQR**(**data**)** ***** length**(**data**)^(-**1**/**3**);**

nrBins **<-** ceiling**(** **(**range**(**data**)[**2**]** **-** range**(**data**)[**1**]** **)** **/** W **)**

entrop **<-** entropy**(** discretize**(**data**,** nrBins**)** **);**

psd **<-** calcPSD**(**data**);**

sec **<-** floor**(**length**(**psd**[**1**,])/**10**)**

LF **<-** list**(**"x"**=**psd**[**1**,**1**:(**sec*****2**)],** "y"**=**psd**[**2**,**1**:(**sec*****2**)]);**

HF **<-** list**(**"x"**=**psd**[**1**,(**sec3**):(**sec*****5**)],**"y"**=**psd**[**2**,(**sec*****3**):(**sec*****5**)]);**

dynamic.range **<-** max**(**LF**$y)-**mean**(**HF**$y);**

AUC.LF **<-** trapz**(**LF**$x,** LF**$y);**

AUC.HF **<-** trapz**(**HF**$x,** HF**$y);**

ratio **<-** AUC.LF**/**AUC.HF**;**

output**[**i**,**1**]** **<-** one.lag**;**

output**[**i**,**2**]** **<-** entrop**;**

output**[**i**,**3**]** **<-** dynamic.range**;**

output**[**i**,**4**]** **<-** ratio**;**

**}**

write.table**(**output**,** file**=**"ictc_feat"**,** quote**=**FALSE**,** col.names**=**FALSE**,** row.names**=**FALSE**);**
